# Where Do Professional Sports Clubs and Organisations Sit Within a Local Health Promotion System? A Social Network Analysis Study

**DOI:** 10.3390/ijerph22020244

**Published:** 2025-02-09

**Authors:** Jack Brazier, Joey Murphy, Charlie Foster, Nick Townsend

**Affiliations:** 1 Centre for Exercise, Nutrition and Health Sciences, School for Policy Studies, University of Bristol, Bristol BS8 1TZ, UK; joey.murphy@bristol.ac.uk (J.M.); nick.townsend@bristol.ac.uk (N.T.); 2 Population Health Sciences, Bristol Medical School, University of Bristol, Bristol BS8 1QU, UK; charlie.foster@bristol.ac.uk

**Keywords:** professional sports clubs, systems, physical activity, health promotion, social network analysis

## Abstract

Professional sports clubs and organisations (PSCOs) have been identified as effective organisations for health promotion (HP); however, their position and connectivity within local HP systems is largely unknown. Our research aimed to explore (i) who identifies PSCOs as a key partner within the delivery of local HP projects, (ii) who they collaborate with, and (iii) their perceived importance by network members. A social network analysis survey was completed by eighteen stakeholders within a South West region of England. Few organisations identified PSCOs as a key partner; however, influential organisations, such as the local authority, reported ties to them. Generally, PSCOs were not identified by network stakeholders as key organisations in the dissemination of HP knowledge or resources, such as project delivery or staffing. Limited relationships with voluntary and health sector organisations existed, and fostering such multisectoral relationships should be a priority for PSCOs in the future. PSCOs were not clearly integrated within the local HP system sampled and are siloed within their local HP activities and delivery. Future research and practice should explore how PSCOs’ assets could be best utilised with local HP and contribute towards local and national health priorities.

## 1. Introduction

Professional sports clubs and organisations (PSCOs) competing with elite sport structures or leagues often adopt corporate social responsibility practices to deliver on social or environmental outcomes [1,2]. Whilst it has been demonstrated that PSCOs as settings can promote unhealthy behaviours, often through sponsorship or affiliation with the gambling or alcohol industry, corporate social responsibility practices often occur through the delivery of health promotion (HP) projects in local communities [3,4,5,6,7,8]. These HP projects commonly offer provisions for people throughout their lives, spanning physical activity (PA) promotion, weight management, mental health support, healthy diets, and many more [3,4,5,6]. Such projects are typically delivered by the PSCOs’ independent charitable organisation affiliated with the club brand, commonly known as ‘Community Trusts’, ‘Foundations’, or ‘[name of club] in the Community’ [3,9]. The presence and capacity of PSCOs within communities has grown significantly over the last 25 years, particularly amongst football PSCOs, whereby ‘Football in the Community’ schemes began to shift from projects that were centred around boosting football participation, to projects that sought to contribute wider health and social policy objectives [10]. The focus on PSCOs contributing to local communities was formally observed in 1986, whereby six ‘community Trusts’/‘foundations’ were founded as part of a pilot scheme developed between the Football League and the Professional Football Association [11]. The initial intention was based upon a need to promote greater relationships between communities and clubs due to fan disorder; however, wider health and social objectives were soon integrated into community trusts and foundations’ work; and the increase in the number of organisations followed [11]. Currently, there are 92 trusts or foundations across the English Football League (EFL) and Premier League, representing all clubs who compete in the elite structure in England and Wales. According to data from the 2021/2022 EFL season, EFL clubs delivered an estimated 579,712 hours of HP or education projects, reaching 840,094 people [11]. In turn, demonstrating their ability to engage with community members at scale.

Previous literature demonstrates that concepts such as fan-attachment to professional sports clubs and the power of PSCO branding can act as ‘pull’ factors which often attract participants to HP projects delivered by PSCOs [12,13,14]. The resources and facilities that PSCOs can access and utilise, such as stadia, coaches linked to the club, and merchandise, can be useful assets for participant retention within HP projects [12,14,15,16]. Moreover, evidence further suggests that PSCOs can engage individuals who are at greater risk of poorer health outcomes or those who may not typically engage with health services, such as, but not limited to, men living with obesity or experiencing mental health conditions [16,17,18,19,20]. Capturing and demonstrating the impact of ‘in-house’ (i.e., internally developed) projects has long been a considerable challenge for PSCOs; however, evidence on large-scale projects designed, supported, or evaluated by academic partners offers insight into the effectiveness of PSCOs as settings for HP [5,6,9]. Specifically, the evaluation of the Premier League Health programme, a men’s HP project funded and delivered by the, at the time, English Premier League Foundation, reported significant increases in participants’ weekly physical activity and consumption of fruit and vegetables, alongside observing significant decreases in daily sitting time and weekly alcohol consumption [21]. Furthermore, a meta-analysis of the Football Fans in Training intervention and the subsequent adapted interventions (studies *n* = 6) report a mean weight loss of −3.3 kg (95% confidence interval: −4.7 to −2.0) at 12 weeks in favour of the intervention [22], whilst also highlighting favourable results for physical activity, sedentary behaviour, dietary markers, psychological wellbeing, health-related quality of life, and cost effectiveness [22].

Despite this, the role of PSCOs within local health and care strategies is unclear. Within the United Kingdom (U.K.), PSCOs linked to football clubs tend to have a greater financial and staffing capacity than PSCOs situated in other sports, often leading to higher levels of HP provision [6]. Amongst these more resourced PSCOs, some have reported commonly working with local stakeholders such as Local Authorities or National Health Service bodies, such as integrated care boards; however, funding constraints are found to often limit project sustainability and, therefore, sustainable strategic collaboration with key local stakeholders [5,6,9]. Subsequently, there is a considerable risk that PSCOs are not strongly embedded into local strategies for health despite their ability to support local community HP, in turn, becoming organisations that are underutilised and siloed within local systems. Given that the responsibility for public health in England was transferred from the National Health Service to Local Authorities in 2013 as part of the Health and Social Care Act in 2012, the exploration of collaboration between PSCOs and local authorities is warranted [23]. Developing effective partnerships between the sport and health sector for local HP can be challenging, particularly when resources are scarce. However, there is an increasing need to explore how these sectors align due to the rising levels of intersectoral reliance required to meet national and local policy objectives within the U.K., such as those reported in Sport England’s Get Healthy, Get Active initiative, and Uniting the Movement Strategy [24,25]. Integral to the delivery of such strategies are active partnerships, of which there are 43 within England [26,27]. Active partnerships are independent not-for-profit organisations that aim to increase PA and reduce inactivity through place-based approaches in their locality [26,27]. Often in receipt of significant funds from Sport England, active partnerships are integral to Sport England’s Uniting the Movement Strategy. Specifically, active partnerships are responsible for providing community-level PA data and insight to Sport England, and are essential in the implementation of the Uniting the Movement Strategy at a local level [28,29]. Moreover, as alluded to, the growing interdependence between the health and sport sector suggests that Active Partnerships will require support from local organisations to deliver on these national strategic and policy goals [25]. Drawing on the available evidence highlighting PSCOs’ ability to recruit participants at risk of future ill health, and the positive impact projects can have upon health outcomes, the exploration of PSCOs’ strategic role within multisectoral approaches to local HP is warranted. Given the need for such multisectoral collaboration in local HP, it is important to understand how local systems operate. In doing so, leverage points, key actors and areas for system development and improvement can be identified. Furthermore, the emerging evidence for the breadth and effectiveness of HP projects delivered by PSCOs, paired with the unique assets and mechanisms for community engagement they hold, underscores the importance of examining the connectedness of PSCOs within local HP systems.

### Context

This research is set in the geographic boundaries of Bristol, North Somerset and South Gloucestershire (BNSSG), a region in the South West of the U.K., populated by approximately one million people [30]. Stark differences in life expectancy mediated by deprivation are reported, whereby women and men living in the least deprived areas are predicted to live between 4.3 and 9.9 years longer than those living in the most deprived wards of the region, respectively, whilst approximately 25,000 children live in poverty [30]. Furthermore, recent data suggest that 31.3% of children are obese or overweight, with higher rates identified amongst black and minority groups, and those living in the poorest areas of the region, whilst 63% of the adult population live with obesity or overweight [30,31]. Such evidence offers insight regarding some of the health priorities, and inequalities, present within the region. To address these, there have been recent calls to action within strategies and reports to develop systems and networks that support multisectoral collaboration, co-production and shared-decision making, to improve health and support healthy behaviours at a community level; particularly for those most impacted by inequity and socioeconomic inequality [32,33]. Moreover, the local Sport and Physical Activity Strategy further emphasises the need for partnership working, suggesting that identifying key partners and networks, and understanding how they can contribute to a system approach for PA promotion in local communities is a priority for the region [34]. Within the region, there are five PSCOs delivering health promoting and educational projects for children and young people, adults, and older adults, all of which hold independent charitable status. Projects are delivered across stadia, schools, sport and leisure venues, and community venues within BNSSG, and mostly use multi-sport models, football, cricket, rugby or basketball to engage and deliver projects for community members. Projects include, but are not limited to, after-school clubs, holiday camps, hybrid classroom-PA sessions, weight management programmes, mental health programmes, inclusion and disability provision, and outsourced school physical education [35,36,37,38]. The five PSCOs operate from three stadia with capacities of 27,000, 11,000, and 8000, reflecting their ability to engage with, or advertise HP projects to, community members and fanbases on matchdays alone. Further opportunities for engaging with community members outside of PSCO stadia can be evidenced through partnerships with schools, whereby reports suggest one organisation partners with 40 primary schools across the region, recruiting over 8000 children to various HP projects and holiday camps throughout 2022/2023 [39]. Moreover, within the region, a PSCO reports reaching over 8000 people in school, community and sport settings, across 16 programmes annually, indicating an age range of 5–104 years old, showcasing the ability of PSCOs within BNSSG to engage with communities in a variety of settings across the region [40]. Given the reported health inequalities and priorities within BNSSG, the identified need for multisectoral collaboration to address these, and active role of five PSCOs operating throughout the region, we argue that this geographical region is of interest to research. Moreover, Bristol is a diversely populated area, and considered a major city within the U.K., yet is not considered a large city by area. In turn, this ensured the research was not only pragmatic and manageable, but externally valid to other cities within the U.K.

Thus, the presence of PSCOs within communities, and the supporting evidence of PSCOs as enablers of HP activity, it is important to better understand the role that PSCOs could play within HP across the region for local policy and practice. Therefore, our study aims to explore the role PSCOs play within a local HP system, and to identify key actors within the system. To answer the aims of the study, we propose to address the following research questions:

(i) Who identifies PSCOs as a key partner in the delivery of HP projects?
(ii) With whom do PSCOs share information, knowledge, and resources?
(iii) What is the perceived importance of PSCOs to the system from other organisations in it?

## 2. Materials and Methods

To address the research aims of this study, a social network analysis (SNA) was undertaken within the geographical boundary of BNSSG. SNA is a research technique often used to explore structural and relational dynamics of health systems and has been used in previous HP and physical activity research, reflecting how actors (e.g., individuals or organisations) interact [41,42]. Directions (e.g., directed and undirected) and types (e.g., mutual) of relations between actors can also be captured, offering insight into real-world social structures and systems [41,43,44].

### 2.1. Participants

A purposive sampling method was adopted to recruit participants. An initial invitation to participate was sent via email to members of a programme board for a collaborative HP programme delivered by multiple PSCOs in the locality. To recruit further, a snowballing technique, commonly used in SNA research, was utilised, whereby participants were able to provide contact details for key connections within partnering organisations [44]. If participants responded with an expression of interest, a participant information sheet and a consent form was provided via email. Informed consent was provided by participants prior to taking part in the research. A total of 65 organisations operating within BNSSG were invited to participate in the study, covering PSCOs (*n* = 5), public (*n* = 9), education (*n* = 3), health (*n* = 5), sport (*n* = 13), Voluntary Community, Faith and Social Enterprise (VCFSE) (*n* = 10) organisations, and primary care networks (*n* = 20). Due to the use of snowball sampling, some organisations listed by participants were not contacted due to falling outside of the geographical network boundary.

### 2.2. Data Collection

Upon providing informed consent, participants were given access to an online survey (Microsoft Forms) that asked participants to list five-to-ten of the most important organisations they work with in delivering health and wellbeing projects across BNSSG. Participants were requested to provide a minimum of five organisations, and for each organisation listed were asked three multiple choice questions regarding the nature of relationship with that organisation. A free-text box was made available for each question to capture wider information. Additionally, participants were asked to specify how frequently they collaborated with each organisation. The survey was developed by JB and reviewed by JM and NT. Iterations included the refinement of questions and addition of free-text boxes. Participants were also invited to participate in a semi-structured interview, which is detailed and reported in a separate paper [45].

### 2.3. Data Analysis

Data gathered via the SNA survey were firstly exported into Microsoft Excel whereby a (1-node) matrix was developed. Data were then cleaned to ensure organisations (nodes) were not duplicated in results. Following this, data were imported to UCINET for analysis whereby tests for centrality and geodesic distance were carried out, and figures were created to visualise findings [46]. Centrality is the overarching term for various individual measures that describe the importance of an organisation/individual (node) within a network [44]. Within our analyses, indegree, betweenness, and ineigenvector centrality were calculated, in addition to an ego network analysis for all PSCOs within the network, and geodesic distance calculations. Given the lack of previous research that has sought to explore PSCOs within HP networks, such measures were deemed the most appropriate to undertake this exploration of PSCOs connectivity, ability to influence other organisations, and the importance other organisations place upon them; offering initial insight concerning PSCOs position within the HP network, and opportunities to develop future partnerships [44].

Indegree centrality provides insight around organisations (nodes) with the most inbound connections reported by other organisations (nodes) within a network or system [44]. In essence, this demonstrates the ‘popularity’ of an organisation (node) [44]. For example, if 10 organisations (nodes) identify Actor A as a partner, and only 5 organisations identify Actor B as a partner, Actor A will have greater indegree centrality. Betweenness centrality represents organisations (actors) within a network that are important for the sharing of information, knowledge or resources [44]. This is calculated by identifying the shortest path, or route, between organisations (nodes), demonstrating the extent to which an organisation can act as a ‘go-between’ for other organisations not directly connected [44]. In turn, highlighting how organisations (nodes) may mediate relationships between other organisations that are not directly connected [44]. Ineigenvector centrality calculates the importance of an organisation (node) within a network, as determined by the relative importance of the organisations (node) that is connected to it [44]. For example, if Actor A and Actor B are highly connected organisations, and both are connected to Actor C, Actor C will have a high ineigenvector centrality. This reflects both the influence that an organisation (node) can have within a network, whether this be on practice or policy, alongside the value that other organisations (node) place upon it. To further explore the direct relationships between PSCOs with the network, and PSCOs’ direct relationships with other organisations within the network, an ego network analysis was undertaken [44]. Geodesic distance was calculated as a measure of cohesion, demonstrating the shortest length of path between two organisations (nodes) within the network, indicating connectivity of a network as a whole [47].

### 2.4. Ethics

Ethical approval for this study was obtained from the University of Bristol’s School for Policy Studies Research Ethics Committee (REF:16169).

## 3. Results

### 3.1. Descriptive Statistics

Eighteen participants completed the SNA survey, resulting in a network of 90 organisations spanning the sport/PA *(n* = 6), public (*n* = 2), and VCFSE *(n* = 3) sectors, alongside primary care networks (PCNs) *(n* = 3), and PSCOs *(n* = 4) (see Table 1). It is important to highlight that PSCOs have been categorised separately due to the scope of this study; however, it should be acknowledged they are positioned in both the sport/PA and VCFSE sector. Moreover, education sector organisations such as schools, colleges and universities, have been distinguished from public sector organisations, whilst the overlap, specifically in relation to schools, is acknowledged, for the purpose of analysis they have been separated to explore connections between different settings.

### 3.2. Indegree Centrality: Who Identifies PSCOs as a Key Partner in the Delivery of HP Projects?

**Figure 1:**
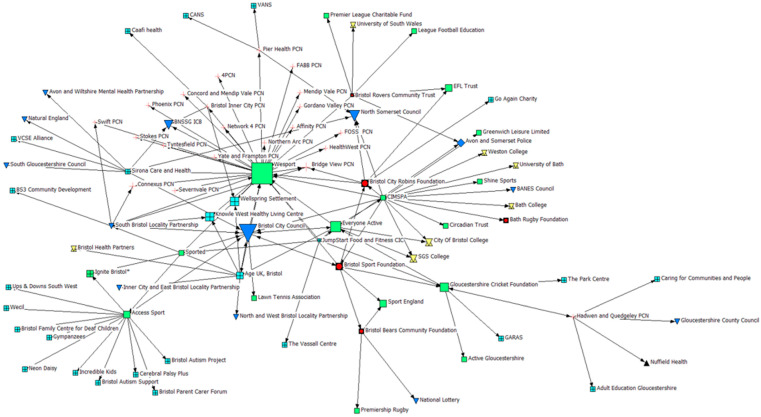
Indegree Centrality. Note: The greater the icon size indicates a higher number of inbound connections.

**Figure 2:**
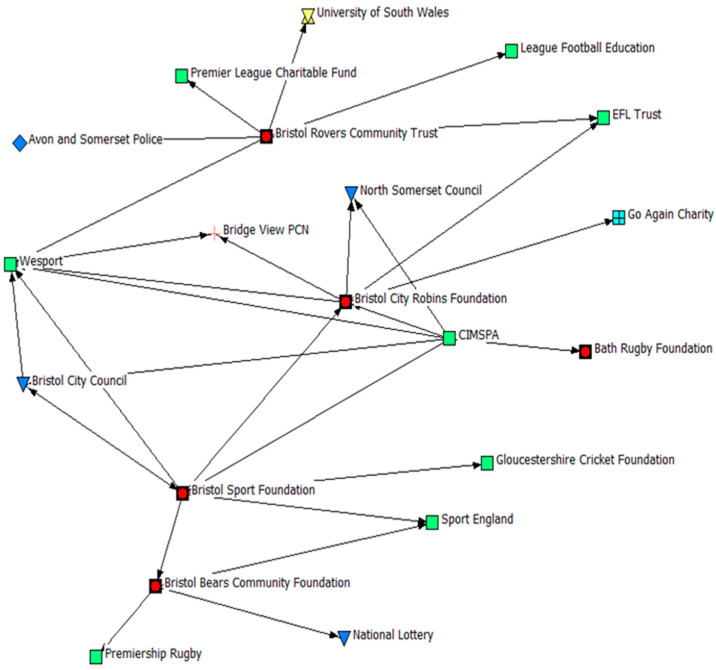
Ego Network of PSCOs within the network. Note: Arrowheads indicate the direction of connections to, or from, PSCOs.

The findings for indegree centrality are visualised in Figure 1, whereby the larger-size icons (node size) reflect a higher level of indegree centrality (i.e., the greater the size, the greater the number of inbound connections). Our analysis suggests that within the network, reported connections to PSCOs within the sample were limited, and therefore they were not organisations that stakeholders viewed as key partners. Limited ties were highlighted between PSCOs within the network, whilst multisectoral ties also appeared low; particularly amongst VCFSE organisations and PCNs. Where connections were reported, CIMSPA, the national body for professional and organisational development in the sport and PA sector, identified multiple PSCOs (*n* = 3/5) as key partners. Moreover, Bristol City Council (Local Authority), a highly connected node, reported the Bristol Sport Foundation (PSCO), who utilise a multi-sport model to provide sport and PA opportunities to children throughout the region, as a key partner. When examining who PSCOs reported as key partners, ties with governing bodies who may provide guidance and funding to PSCOs, such as Sport England and EFL Trust, were commonly identified. To visualise these specific connections with and between PSCOs, an ego network analysis was undertaken (see Figure 2) whereby arrowheads represent an inbound connection. This represents the directional connections between the Bristol Sport Foundation, Bristol City Robins Foundation, Bristol Bears Foundation, Bristol Rovers Community Trust, and the Bath Rugby Foundation, and other organisations in the network.

### 3.3. Betweenness Centrality: With Whom Do PSCOs Share Information, Knowledge, and Resources?

**Figure 3:**
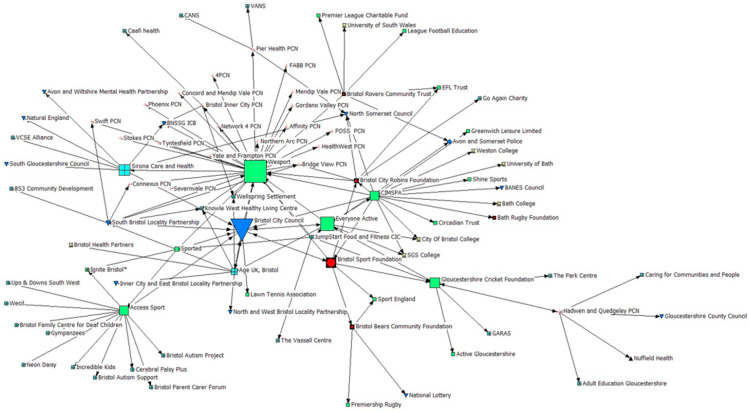
Betweenness Centrality. Note: The greater the icon size represents organisations that are most likely to be important for the sharing of information, knowledge or resources, acting as a ‘go-between’ for unconnected organisations.

Generally, PSCOs within this network were not identified as holding a high level of betweenness centrality, suggesting they may not be influential in the dissemination of materials or knowledge. However, Bristol Sport Foundation reported one of the highest levels of betweenness centrality in the network, and a greater level than that of other PSCOs such as Bristol City Robins Foundation, Bristol Bears Foundation, and the Bristol Rovers Community Trust; suggesting they may have a considerable role within this network in relation to sharing information, knowledge, and resources (see Figure 3). Data suggest Wesport (Active Partnership) and Bristol City Council (Local Authority) are key organisations for dissemination of resources, materials, and knowledge within this network. As the Active Partnership and one of the Local Authorities for the region, they will likely hold knowledge and information surrounding national priorities and directions of travel for sport and physical activity, whilst also holding responsibility for driving place-based agendas for sport and PA, both of which will be of significant importance to PSCOs. In turn, the tie between Bristol City Council and Bristol Sport Foundation may be of particular importance.

Moreover, in light of Bristol Sport Foundation’s ties with both Bristol City Robins Foundation and Bristol Bears Community Foundation, who utilise football and rugby union as a vehicle for HP, respectively, the higher degree of betweenness centrality reflects Bristol Sport Foundations potential capacity to share resources, information or knowledge via partnering PSCOs to wider stakeholders.

### 3.4. Ineigenvector Centrality: What Is the Perceived Importance of PSCOs to the System from Other Organisations in It?

**Figure 4:**
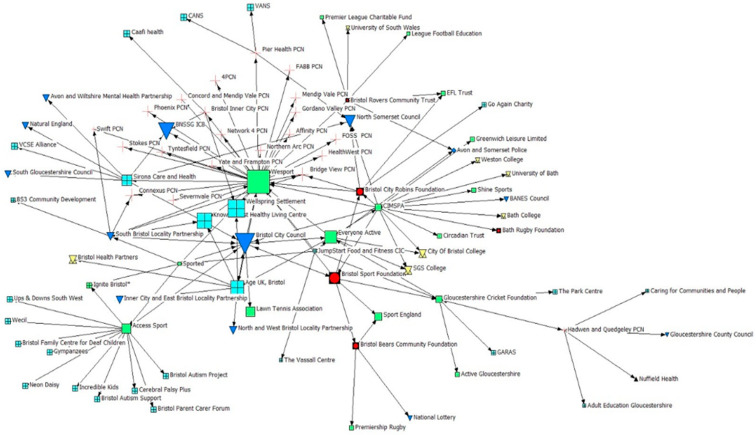
Ineigenvector Centrality. Note: The greater the icon size reflects the greater amount of influence an organisation can have within a network, whether this be on practice or policy, alongside the greater value that other organisations place upon it.

Findings for ineigenvector centrality are visualised in Figure 4, whereby larger icon sizes depict a higher degree of centrality. Findings suggest that Bristol Sport Foundation and Bristol City Robins Foundation (PSCOs) hold moderate ineigenvector centrality, demonstrating that highly connected and influential organisations regarded them as important partners within the network.

Furthermore, consistent with other measures of centrality, Wesport (active partnership) and Bristol City Council (local authority) are shown to be highly important, whilst there is also an increased level of importance identified amongst VCFSE organisations such as the healthy living centres, Age U.K., and Sirona Health and Care. The importance of PCNs is also highlighted within this measure, reflecting their value within this network. Moreover, the importance of public sector organisations such as BNSSG ICB and North Somerset Council are also implied. However, despite the recognised importance of VCFSE organisations and PCNs, the data report that there are limited ties between PSCOs and such organisations. Given the value placed upon VCFSE organisations and PCNs by highly influential organisations such as the local authority and active partnership, further exploration of how PSCOs might be able to collaborate with such organisations, and for what benefit, may be of interest.

### 3.5. Geodesic Distance

To explore the connectivity of the network, calculations for geodesic distance were conducted. An average geodesic distance of 2.966 within this network suggests limited connectivity within the network, reinforcing the influence and importance of key organisations within this network demonstrated by betweenness and ineigenvector centrality calculations (Figure 3 and Figure 4).

## 4. Discussion

Our study aimed to assess the HP system across a South West region of the U.K., exploring the role played by PSCOs and identifying key stakeholders. Our findings suggested that the network as a whole held limited connectivity, emphasising the importance of these organisations within the network. Contextually, qualitative data suggest a historical issue of organisations competing for funding within this network, particularly amongst PSCOs, creating a culture that has restricted collaboration and knowledge sharing [45]. To overcome this, and to develop a more connected and collaborative system, data suggest a commitment to strategic and joint-up thinking at a system level, and working towards local system health priorities, are essential. Whilst recent investment by Sport England under the Local Delivery Pilot and subsequent place partnership initiatives are intended to promote collaboration, and it is a clear ambition within Sport England’s Uniting the Movement Strategy, our data highlight a degree of fragmentation within this system [28,29].

PSCOs themselves were typically not identified as well-connected or influential organisations within this network of local community HP, despite the evidence base suggesting they could be a considerable resource for effective HP delivery within local communities [3,4,5,6,22]. Furthermore, PSCOs are often viewed as community anchor organisations by their fanbases and local residents, whilst also holding the ability to utilise assets such as stadia and branding to engage with communities, furthering the argument for their role within local HP systems [14,48,49]. To our understanding, very little published research has explored the role of PSCOs within local HP system, particularly from a SNA perspective. Qualitative findings from Pringle and colleagues report that PSCOs often communicate and collaborate with local system stakeholders, yet rarely have clearly defined roles with local strategies and struggle to maintain partnerships once initial partnership funding ceases [5,50,51,52]. Viewed in light of our results, PSCOs may be recognised as partners within HP systems, yet our findings suggest that within this network, regardless of PSCOs capacity to act as significant delivery partners and their ability to engage with communities, they are not widely identified as influential or key organisations by system partners. In turn, the lack of connectivity within the network may demonstrate that PSCOs are to a certain degree siloed in their work, and arguably are somewhat underutilised as resources or assets within the system. Despite the lack of connectivity of PSCOs within this network, an increasing interdependency between sport and PA providers, and the health sector exists [25]. Specifically, it is proposed that whilst central and local government, as well as health sector organisations, may hold more power over strategic directions, commissioning and establishing priorities, community sport organisations, such as PSCOs, are essential in determining whether they are able to achieve their policy and strategic objectives [25]. Whilst there is evidence of effective partnership working between PSCOs and statutory health services/providers, whereby PSCOs have been integrated into local healthcare pathways, our findings suggest that within this network such partnerships appear to be lacking [53]. If PSCOs are to be viewed as underutilised, future practice should explore how the ‘pull’ factor of PSCOs and the delivery capacity can be maximised, not only within this network but nationally.

Within this network, understanding organisational strengths, weaknesses and assets, and working towards shared priorities, will be essential for more effective and sustainable efforts to promote local community health [45]. Similar recommendations for multisectoral collaboration within local HP efforts have been reported, suggesting that agreement upon objectives, roles and responsibilities must be reached by all stakeholders to facilitate effective collaboration, and to develop future partnership working [54]. We propose that local decision makers and commissioners should explore how PSCOs’ capacity to deliver projects in, and engage with, local communities can be better utilised within systems approaches to HP, to support addressing local health priorities outlined within strategic documents, such as those produced by Local Authorities’ Public Health departments or local National Health Service bodies. An example of such opportunity within this network can be found through local calls for whole-system approaches to obesity prevention in response to high rates of obesity reported in this geography [30,31,33]. The exploration of PSCOs role as not only providers of community PA and sport, but also their ability to reduce unhealthy behaviours or commodities within stadia may be of interest to addressing such aims [7,8]. Moreover, local policymakers, decision makers, and practitioners across sectors should collaboratively develop local priorities for HP and develop a clearer understanding of organisational strengths and gaps within the network.

Bristol City Council (local authority) and Wesport (active partnership) were identified as the most connected and influential organisations within the network, demonstrating their significant role and responsibility for HP. Local authorities are often leaders, or at least central, to many local and regional networks and partnerships in health and care, whilst also having the capacity to influence services concerning the wider determinants of health, such as housing or transport [23]. Leader organisations within systems and networks hold responsibility for bringing organisations together, identifying shared goals and benefits, and aligning work to national objectives and priorities [27]. Whilst this is a challenging task, evidence suggests it can be an effective mechanism for shifting from traditionally adopted medical models of health, into more sustainable systems approaches with devolved power and leadership, which, given the increasing need for collaboration between the health and sport sector, may be required in this context [25,55,56]. Sport England’s Uniting the Movement Strategy and associated 2022–2025 Implementation Plan highlight these organisations as crucial in facilitating local, collaborative place-based approaches to improving health and reducing inequalities through PA and sport [28,29]. Moreover, within Sport England’s commitment to expand their place-based partnerships, active partnerships play an essential role in fostering and maintaining long-term strategic partnerships with organisations with ‘trust’, ‘credibility’ and ‘connections’ within communities [26]. Utilising the ‘pull’ of PSCOs, namely branding and community presence, therefore may be highly influential in active partnerships’ ability to support the delivery of PA promotion strategies. However, our findings suggest that partnerships between PSCOs, local authorities and active partnerships may be underdeveloped. Therefore, to support effective delivery of this strategy, particularly around the engagement of those at risk of future ill-health and those who do not traditionally engage with services, we propose that PSCOs may be important organisations nationally for active partnerships and local authorities to consider their relationships with and should both explore how PSCOs could support local and national PA and sport, and wider HP goals.

### Limitations

When considering our findings, it is important to acknowledge the limitations of the study. Firstly, due to the focus of the paper on PSCOs, and the specific locality of the network, findings are based upon a small local network, and therefore may not be generalisable to other parts of the U.K. However, our findings offer insight for future practice and development of the network within this locality, whilst also offering recommendations for practice, and a replicable model for research to take place, not only in other Sport England-funded local delivery pilot areas, but local area approaches to HP nationally and internationally. Moreover, the limitations of SNA as a method should be acknowledged, whereby a near-to-completed network consisting of all active members should be sought [47]. Whilst we did not receive responses from all 90 organisations included in the sample, 80% of PSCOs within the boundary provided survey responses. Moreover, we did not receive survey responses from organisations within the education sector and received few responses from PCNs and other health providers, thus limiting the scope of the network from these perspectives. In attempts to negate this limitation, multiple attempts were made to contact all organisations listed as key partners within survey responses utilising contacts provided within survey responses, publicly available methods (e.g., enquiry inboxes and social media platforms), and community of practice networks. However, not all organisations responded, and some declined the opportunity to participate, possibly due to lack of time, capacity, or perceived lack of relevancy. It should therefore be considered that our findings may not be a true representation of the network as a whole, but do offer novel insight regarding PSCOs position within the network, alongside recommendations for future practice and research both within and beyond this network. Future research should consider alternative, or supplementary, approaches to data collection, such as stakeholder mapping workshops or focus groups, to ensure representation across all sectors. Given the sample within our study is not powered to fully explore potential healthcare or school-based opportunities for PSCOs, such approaches that may boost recruitment may help to identify future collaboration between PSCOs and the health and education sectors.

## 5. Conclusions

Our study aimed to explore where PSCOs fit within local HP delivery and strategy via SNA. Our findings report that PSCOs were not well connected or integrated organisations within this network, nor did they hold a significant level of influence amongst other organisations. Moreover, the connectivity of the network as a whole was limited, and therefore places considerable responsibility upon local authorities and the local active partnership to develop a more cohesive network. Despite the ability of PSCOs to deliver a high provision of HP projects, their status within communities, and their capacity to engage members, their role within local delivery and strategy appears unclear and somewhat siloed. Future practice should engage in further stakeholder and asset mapping within networks such as (1) exploring the potential role, delivery and assets PSCOs could offer a system, (2) enabling best use of limited resources in tackling local and national health priorities, and (3) advocating for national guidance and support for facilitating strategic joint-up working, such as funding opportunities that promote partnerships and collaboration locally.

## Figures and Tables

**Table 1:** Descriptive statistics of survey respondents and network.

Organisation Classification	% of Survey Respondents	% of Network	Representation in Figures
PSCO	22.2% (*n* = 4)	5.6% (*n* = 5)	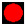
Sport/PA Sector	33.3% (*n* = 6)	18.9% (*n* = 17)	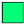
Public Sector	11.1% (*n* = 2)	13.3% (*n* = 12)	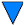
VCFSE Sector	16.7% (*n* = 3)	28.9% (*n* = 26)	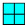
Education/Research Sector	0% (*n* = 0)	7.8% (*n* = 7)	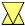
PCN/Health	16.7% (*n* = 3)	24.4% (*n* = 22)	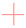
Other	0% (*n* = 0)	1.1% (*n* = 1)	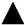

PSCO: Professional Sports Club or Organisation; VCFSE: Voluntary, Community and Faith groups, and Social Enterprises; PCN: Primary Care Network.

## Data Availability

Data can be requested through contacting the corresponding author.

## Abbreviations

The following abbreviations are used in this manuscript:

PSCO	Professional Sports Club and Organisations
HP	Health Promotion
PA	Physical Activity
EFL	English Football League
U.K.	United Kingdom
BNSSG	Bristol, North Somerset, and South Gloucestershire
SNA	Social Network Analysis
VCFSE	Voluntary, Community, Faith and Social Enterprise
PCN	Primary Care Network
